# CMBF: Cross-Modal-Based Fusion Recommendation Algorithm

**DOI:** 10.3390/s21165275

**Published:** 2021-08-04

**Authors:** Xi Chen, Yangsiyi Lu, Yuehai Wang, Jianyi Yang

**Affiliations:** College of Information Science and Electronic Engineering, Zhejiang University, Hangzhou 310063, China; chen__xi@zju.edu.cn (X.C.); luyangsiyi@zju.edu.cn (Y.L.); yangjy@zju.edu.cn (J.Y.)

**Keywords:** recommendation systems, multi-modal algorithm, cross-modal fusion, attention mechanism

## Abstract

A recommendation system is often used to recommend items that may be of interest to users. One of the main challenges is that the scarcity of actual interaction data between users and items restricts the performance of recommendation systems. To solve this problem, multi-modal technologies have been used for expanding available information. However, the existing multi-modal recommendation algorithms all extract the feature of single modality and simply splice the features of different modalities to predict the recommendation results. This fusion method can not completely mine the relevance of multi-modal features and lose the relationship between different modalities, which affects the prediction results. In this paper, we propose a Cross-Modal-Based Fusion Recommendation Algorithm (CMBF) that can capture both the single-modal features and the cross-modal features. Our algorithm uses a novel cross-modal fusion method to fuse the multi-modal features completely and learn the cross information between different modalities. We evaluate our algorithm on two datasets, MovieLens and Amazon. Experiments show that our method has achieved the best performance compared to other recommendation algorithms. We also design ablation study to prove that our cross-modal fusion method improves the prediction results.

## 1. Introduction

The rapid development of the information age is a double-edged sword which not only brings convenience to people’s lives but also brings us troubles such as data flooding problem. Imagine that when we just want to choose a movie for entertainment without a clear goal, we will be likely to become lost in countless movies. We need an “expert” that can automatically analyze our historical interests and find the specific movie meeting our individual needs from a large library of movies. This “expert” is the recommendation system [1].

Recommendation systems typically record the preference of a user according to the user’s historical operation, and then recommend according items that the user may also like. In the recommendation system, the number of users and recommended items is large, but the number of actual interactions between the users and items is usually rare, although the interaction data are important for recommendation. Many experts have made many efforts to solve the sparsity problem of the interaction data. Some studies [2,3] indicate that a content-based recommendation algorithm can calculate the similarity of the users or items through characteristics to ease the data sparsity problem. For example, Gunawardana and Meek [4] use Boltzmann Machines to model the relationship between the users and items. They bound the parameters of the model with content features to solve the data sparsity problem. Later, more content-based algorithms using deep neural networks are proposed to learn the content features more effectively [5,6,7,8]. However, these algorithms cannot work well when the original characteristics of the users and items are not enough.

The importance of image features in recommendation systems: Recently, multi-modal technologies have been used in recommendation systems [9,10,11] to extend the properties of the users and items by using complementary information such as images. The image feature of the item provides some information that is not available in the text, which has an impact on the user’s preference. For example, one would not buy a t-shirt from Amazon without seeing an image of the item [12]. In addition, in movie recommendation, the user can obtain an more intuitive understanding of the movie through the images, which can help to determine whether he/she is interested in the movie. Therefore, incorporating image features into the recommendation factors can have a positive impact on the accuracy of the recommended results.

Most of the existing multi-modal recommendation algorithms use the same feature learning method and do not additionally learn the relationship between different modal features, which could lose some fine-grained information and affect the prediction results.

In this paper, we propose a Cross-Modal-Based Fusion Recommendation Algorithm (CMBF) to alleviate the data sparsity problem mentioned above. Our algorithm can capture both the single-modal feature and the cross information between two modal features. The CMBF contains four modules: the preprocessing module, single-modal learning module, cross-modal fusion module and output module. Firstly, the image and text features are extracted and embedded in the preprocessing module. Next, we use the single-modal learning module to learn the feature of the single modality. The single-modal features are then fed into the cross-modal fusion module to learn the cross-modal features between two modalities. Finally, we fuse both the single-modal features and the cross-modal features to obtain the high-level feature and predict the recommendation results. An attention mechanism is used in our algorithm to extract more meaningful feature. We also adopt residual connection to combine different levels of feature information.

To summarize, in this paper we make the following contributions:

- We find the problem that insufficient fusion of multi-modal features will lead to the loss of some fine-grained information and affect the prediction results;
- We propose a novel cross-modal fusion method to fuse the multi-modal features completely and learn the cross information between different modalities;
- We evaluate our algorithm on two datasets, MovieLens and Amazon. Experiments show that our method has achieved the best performance compared to other recommendation algorithms.

The rest of the paper is organized as follows. Section 2 presents the problem definition of the recommendation system and presents the related works. Section 3 presents the model architecture of the CMBF. Section 4 presents the experiments and results on the MovieLens dataset and Amazon dataset. Section 5 concludes this paper and points out the future work.

## 2. Related Work

People can gain information from images, sounds, texts and so on. In other words, our world is a multi-modal world. When a research question or dataset contains multiple modalities, it can be processed by multi-modal technologies. Multi-modal technologies can be applied in various fields. For example, one of the earliest applications of multimodal research is audiovisual speech recognition (AVSR) [13], which uses visual information to improve the accuracy of speech recognition. Multi-modal technologies have also played an important role in emotion recognition [14], image description [15], VQA [16], traffic event detection [17] and other fields.

Using multi-modal technologies in the recommendation system can alleviate the data sparsity problem and optimize the performance of the recommendation system. In 2007, Microsoft’s Yang et al. [18] first proposed the concept of multi-modal recommendation. They use the three-modal information of text, image and audio as input to calculate the similarity of between each pair of modals separately, and then use Attention Fusion Function for fusion. Although the feature extraction of the three modalities is very rough at that time, this article still plays a milestone role in the research of multi-modal recommendation algorithms, and the subsequent work can be expended on this basis. For instance, Oramas et al. [19] use the artist’s text to describe information and audio track information to solve the cold start problem in music recommendation. They aggregate all the songs of the same artist to learn the characteristics of the artist, then learn the music feature information for the sound track, and stitch them as the input of a multi-modal fusion network. Finally they get the fusion representation feature of the entire music; Cai et al. [9] of Youku proposed a multi-view active learning framework for video recommendation, which extracts missing text information from visual information to obtain more training videos; Ge et al. [10] believe that the use of user behavior images to enhance behavior representation is helpful to understand the user’s visual preferences and greatly improve the accuracy of prediction. Therefore, they propose to jointly model user preferences with user behavior ID characteristics and behavior images; Wu et al. [11] proposed a multi-view news recommendation based on the attention mechanism. They regard information such as headlines, texts, and subject classifications as multiple forms of news. They encode different forms of information, and then use the attention mechanism to fuse them. These multi-modal recommendation methods usually extract the feature of single modality and simply concatenate the features of different modalities to predict the recommendation results. Obviously, the simple concatenation cannot fuse the multi-modal features completely which may cause the loss of important information. Therefore, we propose a new fusion method called cross-modal fusion to fully fuse the multi-modal features and achieve fine-grained information for prediction. The details of the cross-modal fusion method are presented in Section 3.5.

## 3. Model Architecture

### 3.1. Problem Definition

In a classic recommendation system, we define a user set $U=\{u_{1},u_{2},\ldots ,u_{N}\}$ that represents *N* users, where $u_{i}$ represents the *i*-th user. The feature of each user *u* can be described by a feature vector $X_{u}=\left\{x_{1},x_{2},\ldots ,x_{P}\right\}$, where $x_{j}$ represents the *j*-th feature and *P* represents the total feature number. Similarly, we can also define an item set $V=\{v_{1},v_{2},\ldots ,v_{M}\}$ to represent M items, with the feature vector $X_{v}=\left\{x_{1},x_{2},\ldots ,x_{Q}\right\}$ representing the feature of each item *v*. The interaction matrix between the user and the item is defined as $Y=\{y_{uv}|u\in U,v\in V\}$, where $y_{uv}$ represents the interaction of the user *u* with the item *v* obtained according to the user’s implicit feedback, such as clicking, browsing, purchasing and scoring. The value of $y_{uv}$ is set to 1 when the interaction between the user u and the item *v* is observed, otherwise the value of $y_{uv}$ is 0. The goal of the recommendation system is to infer the possible interaction ${\hat{y}}_{uv}$ between the user *u* and the item *v* that have not been shown before.

### 3.2. Overview

**Figure 1:**
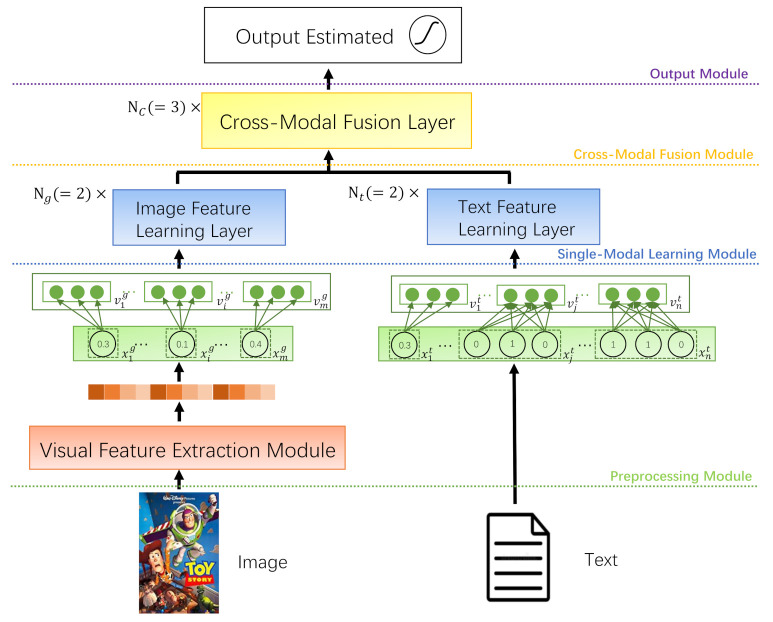
Overview of the proposed framework based on CMBF. The details of the Image/Text Feature Learning Layer and the Cross-modal Fusion Layer are illustrated in Figure 2 and Figure 3, respectively.

Our CMBF framework can be divided into four parts, as shown in Figure 1. Firstly, we extract the image and text feature of users and items and encode them through different embedding methods in the preprocessing module. Then, the dimensionality-reduced Image Embedding Vectors and Text Embedding Vectors are sent to the single-modal learning module, respectively. Next, the interaction and fusion of the two modalities is performed in the cross-modal fusion module to learn the high-level feature representation. Finally, in the output module, the output of the cross-modal fusion module will be further used to predict the recommendation result. The specific implementation details will be expanded in subsequent sections.

### 3.3. Preprocessing Module

Firstly, we should extract the image and text feature of the users and items, respectively. As shown in Figure 1, the Visual Feature Extraction Module (generally CNN-based) is used to obtain the item’s image feature. The image feature vector of the item *v* can be denoted as

(1) $$x^{g,v}=\left\{x_{1}^{g,v},x_{2}^{g,v},\ldots ,x_{m}^{g,v}\right\},$$

where *g* represents ’image’, and *m* is the length of the image feature vector $x^{g,v}$.

Since the text features include the numerical category and the classification category, we use different methods to obtain the text feature. As shown in Figure 1, we use the specific value directly as the corresponding value of the numerical text feature and use the binary representation of “0/1” to represent the categorized text feature. For example, if the price of an item is “10.5$”, the text feature of the price is “10.5”; if the brand of the item is “Fossil”, the value of the “Fossil” is “1” in the text feature of the brand. Then we can obtain the text feature vector of the item *v* and the user *u*, respectively as

(2) $$x^{t,v}=\left\{x_{1}^{t,v},x_{2}^{t,v},\ldots ,x_{v_{n}}^{t,v}\right\},\;x^{t,u}=\left\{x_{1}^{t,u},x_{2}^{t,u},\ldots ,x_{u_{n}}^{t,u}\right\},$$

where *t* represents ’text’, $v_{n}$ and $u_{n}$ represent the length of the text feature vector $x^{t,v}$ and $x^{t,u}$, and $v_{n}+u_{n}=n$, *n* is the total length of the text feature vector. We combine all the image and text feature, respectively, to obtain the final representation of the two modal feature vectors as follows:

(3) $$x^{g}=x^{g,v}=\left\{x_{1}^{g},x_{2}^{g},\ldots ,x_{m}^{g}\right\},\;x^{t}=\mathrm{concat}\left\{x^{t,v},x^{t,u}\right\}=\left\{x_{1}^{t},x_{2}^{t},\ldots ,x_{n}^{t}\right\}.$$

To alleviate the high dimensionality and sparseness of the feature vectors, we use the following methods proposed in [7] to perform embedding dimensionality reduction on the numerical feature, single-value classification feature, and multi-value classification feature:

(4) $$v_{i}=\left\{\begin{array}{cc} w_{i}x_{i}, & \mathrm{for}\;\mathrm{numerical}\;\mathrm{features},\\ W_{i}x_{i}, & \mathrm{for}\;\mathrm{single}-\mathrm{value}\;\mathrm{classification}\;\mathrm{features},\\ \frac{1}{Q}W_{i}x_{i}, & \mathrm{for}\;\mathrm{multi}-\mathrm{value}\;\mathrm{classification}\;\mathrm{features}, \end{array}\right.$$

where $x_{i}$ is the element in the image or text feature vector, $v_{i}$ represents the reduced-dimensional embedding vector, $w_{i}$ represents the Embedding Mapping Vector in the case of the numerical feature, $W_{i}$ represents the Embedding Mapping Matrix in the case of the classification feature, and *Q* represents the number of all potential values if $x_{i}$ is a multi-value feature vector.

After the image feature vectors and the text feature vectors are both encoded as Equation (4), the Image Embedding Vectors and the Text Embedding Vectors will be obtained and prepared for the next module.

### 3.4. Single-Modal Learning Module

The multi-head self attention mechanism [20] can help to learn subtle feature information in the single-modal feature, and each “head” can be regarded as a subspace. Taking the text feature as an example, in the *h*-th feature subspace, the similarity between the Text Embedding Vector $v_{i}$ and the Text Embedding Vector $v_{j}$ can be calculated as

(5) $$\theta^{\left(h\right)}\left(v_{i},v_{j}\right)=\mathrm{Similarity}\left(W_{Query}^{\left(h\right)}v_{i},W_{Key}^{\left(h\right)}v_{j}\right),$$

where $W_{Query}^{\left(h\right)}$ and $W_{Key}^{\left(h\right)}\in R^{d^{{}^{\prime }}\times d}$ are the transformation matrix that maps the embedding vectors from the original space $R^{d^{{}^{\prime }}}$ to the space $R^{d}$. Then the weighted representation of $v_{i}$ in the *h*-th feature subspace is

(6) $${\tilde{v}}_{i}^{\left(h\right)}=\sum_{k=1}^{n}\alpha_{t,i,k}^{\left(h\right)}\left(W_{Value}^{\left(h\right)}v_{k}\right)=\sum_{k=1}^{n}\frac{exp(\theta_{t}^{\left(h\right)}\left(v_{i},v_{j}\right))}{\sum_{k=1}^{m}exp\left(\theta_{t}^{\left(h\right)}\left(v_{i},v_{k}\right)\right)}\left(W_{Value}^{\left(h\right)}v_{k}\right).$$

In Equation (6), $\alpha_{t,i,k}^{\left(h\right)}$ is the normalized weight of $\theta_{t}^{\left(h\right)}\left(v_{i},v_{j}\right)$, $W_{Value}^{\left(h\right)}\in R^{d^{{}^{\prime }}\times d}$ is the transformation matrix, and *m* is the total number of the text feature number. By splicing all the subspaces and using residual connection, we can obtain the new representation of the text embedding vector:

(7) $$v_{i}^{Res}=\mathrm{ReLU}\left({\tilde{v}}_{i}+W_{Res}v_{i}\right),$$

where $v_{i}$ is the concatenation of $\left\{{\tilde{v}}_{i}^{\left(1\right)},{\tilde{v}}_{i}^{\left(2\right)},\ldots ,{\tilde{v}}_{i}^{\left(H\right)}\right\}$, *H* is the total number of the subspaces, and $W_{Res}\in R^{Hd^{{}^{\prime }}\times d}$ is the mapping matrix.

**Figure 2:**
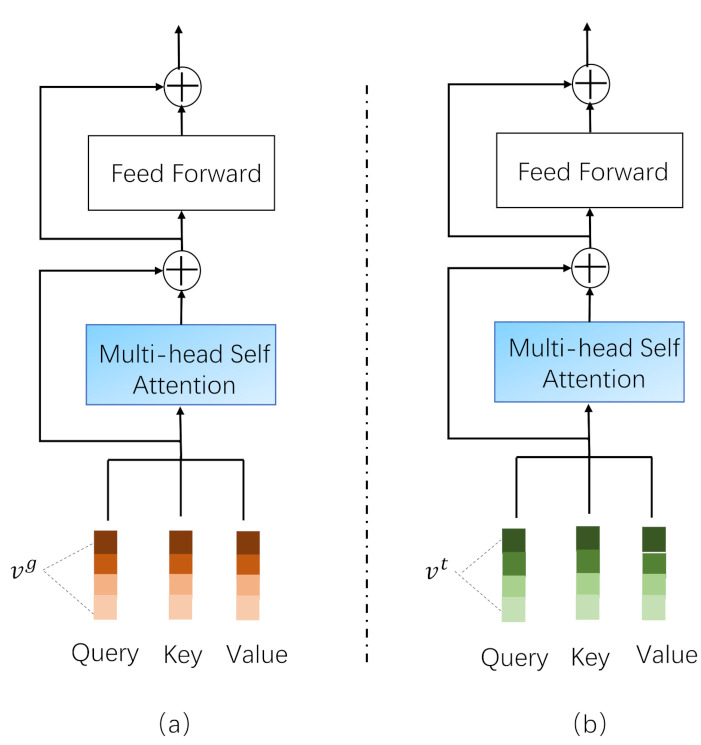
Illustration of the Feature Learning Layer. (**a**) Represents the Image Feature Learning Layer and (**b**) represents the Text Feature Learning Layer.

As shown in Figure 2, the operation through Equations (5)–(7) is called a feature learning layer which can get feature representation of the text or image. We stack multiple feature learning layers to obtain a single-modal feature learning module, so as to obtain the representation of the single-modal feature.

### 3.5. Cross-Modal Fusion Module

In addition to the single-modal feature, the relationship between the text modal feature and the image modal feature of items can also be obtained by data mining and used as supplementary information to further alleviate the data sparse problem of the recommendation algorithm. This is also one of the problems that need to be solved in multi-modal learning technology [21].

Compared with existing multi-modal recommendation algorithms that simply splice single-modal recommendation results, we try to exchange the feature information of the two modalities before fusing these two modal features, and then continue to learn high-level representations of feature. As this feature information exchange operation is similar to a mutual reference of feature across two modalities, we call it “cross-modal fusion”.

**Figure 3:**
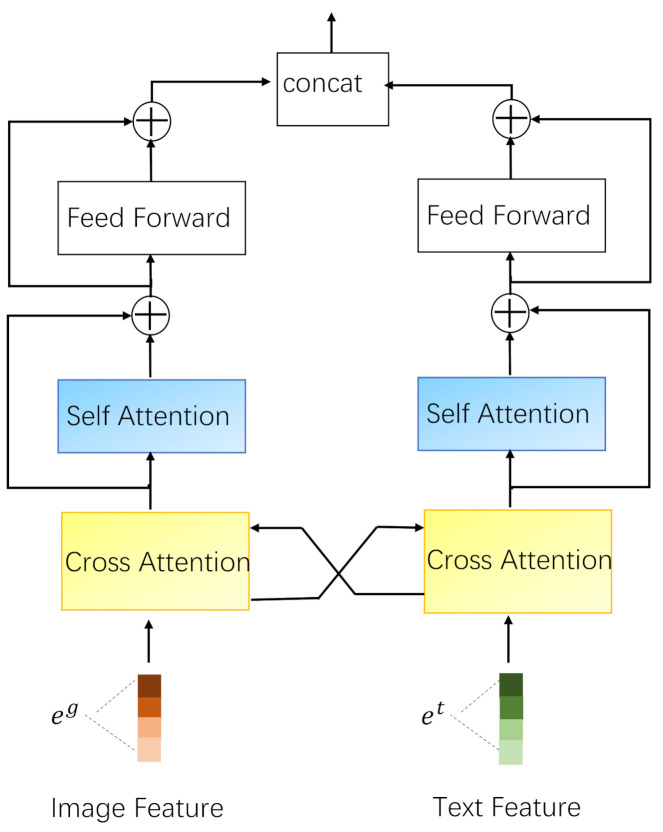
Illustration of the Cross-modal Fusion Layer.

We denote the output of the text feature and the image feature after their respective single-modal learning modules as: $e^{t}$ and $e^{g}$. The cross-modal fusion layer is composed of two cross attention layers and two self-attention [20] layers as shown in Figure 3. We use the image feature $e^{g}$ to calculate the cross-modal weight $\alpha_{k}^{g\to t}$ of the text feature $e^{t}$, and obtain the text cross representation $e_{Cross}^{t}$ using $\alpha_{k}^{g\to t}$ and $e^{t}$:

(8) $$\alpha_{k}^{g\to t}=\mathrm{softmax}\left(\frac{e_{k}^{g}{e_{k}^{t}}^{T}}{\sqrt{d_{e_{k}^{t}}}}\right),$$

(9) $$e_{Cross}^{t}=\sum_{k=1}^{n}\alpha_{k}^{g\to t}e_{k}^{t}.$$

In Equation (8), softmax(·) is a normalization function, $d_{e_{k}^{t}}$ is the dimension of $e_{k}^{t}$, and $e_{k}^{g}{e_{k}^{t}}^{T}$ represents that the relationship between two modal feature is learned through the inner product. In the same way, we can use the text feature $e^{t}$ to calculate the cross-modal weight $\alpha_{k}^{t\to g}$ of the image feature $e^{g}$, and obtain the image cross representation $e_{Cross}^{g}$ using $\alpha_{k}^{t\to g}$ and $e^{g}$:

(10) $$\alpha_{k}^{t\to g}=\mathrm{softmax}\left(\frac{e_{k}^{t}{e_{k}^{g}}^{T}}{\sqrt{d_{e_{k}^{g}}}}\right),$$

(11) $$e_{Cross}^{g}=\sum_{k=1}^{n}\alpha_{k}^{t\to g}e_{k}^{g}.$$

Then we send the text cross representation $e_{Cross}^{t}$ and the image cross representation $e_{Cross}^{g}$ to the self-attention layer and use the residual connection to maintain the previous feature information. To obtain the higher-level information, we stack multiple cross-modal fusion layers to calculate the relationship between the two modal features, and finally concatenate all the feature vectors for the subsequent prediction.

## 4. Experiments and Results

### 4.1. Evaluated Datasets

The datasets used in this experiment are the MovieLens dataset [22] and Amazon dataset [23]. The MovieLens dataset is a public movie data set, which contains multiple users’ rating information for different movies, as well as user and movie characteristics information. It has multiple data scales according to the number of ratings, and we use the movie dataset MovieLens-1M with a sample size of about 1M in this paper. The Amazon Product dataset is a public electronic product dataset, which contains Amazon product reviews and metadata, as well as user ratings and reviews of products. We use a subset of Clothing & Shoes & Jewelry for experimental testing. The introduction of the two data sets is shown in Table 1.

Both datasets lack relevant image information, but related download links are provided in the original data. That is, the MovieLens dataset provides download links for movie posters, and the Amazon dataset provides download links for product images. We use crawler technologies to obtain the corresponding image of each movie or product, and then we use the InceptionV3 model [24] pre-trained on ImageNet (ILSVRC2012) as the image feature extraction module to extract image features from these pictures. In this experiment, we use the 2048-dimensional features by the third pooling layer of InceptionV3 as the image feature for subsequent model calculations.

The processed attributes of the MovieLens dataset and Amazon dataset are shown in Table 2 and Table 3, respectively. For uncommon attributes which appear less than a certain threshold, we mark them as a single attribute as “unknown”. In the MovieLens dataset and the Amazon dataset, the thresholds are 10 and 5, respectively. Specifically, for the attributes of movie year, we treat every ten years from 1919 to 1970 as an attribute, every year after 1970 as an attribute, and the rest of the years as “unknown” attribute. In the two datasets, the user’s score ranges from 0 to 5 points, so we should binarize the scoring information of the original dataset as classification labels. Therefore, scores greater than 3 are regarded as positive samples and scores below 3 are considered negative. We eliminate neutral samples with scores equal to 3 points. In addition, we divide the entire dataset into a training set, validation set and test set according to the ratio of 8:1:1 for experiments.

### 4.2. Experimental Setup

#### 4.2.1. Competing Algorithms

We have divided all the competing algorithms into three categories. The first category is the classic algorithm used before the advent of deep networks:

- **LR** [25] is a commonly used model for recommendation tasks before the deep network is proposed.
- **FM** [26] simulates the importance of the first-order feature and the interaction of the second-order feature.

The second category is the single-modal recommendation algorithm implemented using deep networks:

- **DeepFM** [27] is an end-to-end model using a joint decomposition machine and a multi-layer perceptron. It uses deep neural networks and factorization machines to model the interaction of high-order feature and the interaction of the low-order feature, respectively.
- **Wide&Deep** [28] is a hybrid model composed of a single-layer Wide part and a multi-layer Deep part. The main function of the Wide part is to give the model a strong “memory ability”; the main function of the deep part is to give the model a “generalization ability”, so that the model has both the advantages of logistic regression and deep neural network.
- **AutoInt** [7] is an encoder that automatically learns high-order feature combinations of input feature. It maps digital features and classification features to the same low-dimensional space and uses a self-attention mechanism to learn the interaction of the feature in the low-dimensional space.
- **MiFiNN** [29] calculates the mutual information of each sparse feature and the click result as the weight of each sparse feature. It constructs an interactive method combining the outer product and inner product to carry out the feature interaction.
- **ADI** [30] captures the latent interest sequence in the interest extractor layer, and employs auxiliary losses to produce the interest state with deep supervision.

The third category is the recommendation algorithm based on multi-modal fusion:

- **VBPR** [12] integrates visual information into the prediction of people’s preferences. Compared with the matrix factorization model that only relies on the hidden vector of the user and the item, VBPR is greatly improved.
- **MLFM** [11] is a multimodal late fusion classification method based on text and image. They use machine learning models to extract text and image features, learn a specific classifier for each modal, and then learn a fusion strategy from the results of each modal classifier.
- **DICM** [10] combines user preferences with user behavior ID characteristics and behavior images. This method merges the user’s historically visited item pictures and candidate item pictures using an attention mechanism, and finally fully interacts the traditional ID feature, candidate item image information, and the user’s historical visual preferences to obtain the final prediction result.

#### 4.2.2. Parameter Settings

We use an Adam optimizer [31] with $lr=0.0001,\;\beta_{1}=0.9,\;\beta_{2}=0.999$, and set the batch size as 1024, the dimension of the embedding vector as 16. In the single-modal learning module, the number of the image feature learning layers is denoted as $N_{g}$ and the number of the text feature learning layers is denoted as $N_{t}$. In the cross-modal fusion module, the number of the cross-modal fusion layers is denoted as $N_{c}$. Following AutoInt [7], we set $N_{g}=N_{t}=2$. After experiments, we find that the performance of CMBF is best when $N_{c}=3$. The details of the parameter ablation experiment are described in Section 4.4.1. In each layer, the number of attention heads is two.

#### 4.2.3. Evaluation Metrics

We use Logloss, AUC, GAUC as the evaluation metrics. The calculation formula of Logloss is as follows:

(12) $$\mathrm{Logloss}=-\frac{1}{T}\sum_{t=1}^{T}\left(y_{t}log\left({\hat{y}}_{t}\right)+\left(1-y_{t}\right)log\left(1-{\hat{y}}_{t}\right)\right),$$

where $y_{t}$ and ${\hat{y}}_{t}$ are the real and predicted interaction, respectively, *T* is the total number of the samples in the training set. AUC (Area Under Curve) is a metric which can evaluate the performance of the classification algorithm. However, AUC does not treat different users differently. Then users who do not click on any advertisements may make the AUC results tend to be lower. Therefore, we additionally use GAUC (Group AUC) metric proposed by Alibaba’s Gai et al. [32] to evaluate the model. GAUC can be considered as a weighted average of the AUC of all users:

(13) $$\mathrm{GAUC}=\frac{\sum_{u\in U}w_{u}\times {\mathrm{AUC}}_{u}}{\sum_{u\in U}w_{u}},$$

where $w_{u}$ is the number of operations of the user *u*, $AUC_{u}$ is the AUC value of the user *u*.

### 4.3. Results and Analysis

The experiment results on the MovieLens dataset are shown in Table 4. Our algorithm CMBF achieves the best performance compared to other recommendation algorithms. The performance of CMBF is much better than the classic algorithms (i.e.; LR and FM) since LR and FM cannot capture non-linear relations of information. Compared with the single-modal models (i.e.; DeepFM, Wide&Deep and AutoInt), CMBF uses multi-modal information to alleviate data sparsity and better capture user preference information for more personalized recommendations. The difference between the CMBF algorithm and other multi-modal algorithms (i.e.; VBPR, MLFM and DCIM) is that CMBF exchanges the feature information of the two modalities before fusing the two modal features. The original representation of users and items include both single-modality and multi-modality, and now we have added cross-modal information to the representation of users and items through the cross-modal module, so that we can use the learned relationship between the two modal features to further enrich the data and alleviate the problem of data sparseness.

The experiment results on the Amazon dataset are shown in Table 5. The overall effect of all methods on the Amazon dataset is not as good as that on the MovieLens dataset. The reason is that the sparsity of the Amazon dataset is much higher than that of the MovieLens dataset. Therefore, high sparsity is indeed a key reason that limits the performance of recommendation algorithms, and it is worthwhile to continue to be explored.

### 4.4. Ablation Study

#### 4.4.1. Influence of Parameters

In this section, we study the contribution of cross-modal fusion module to the final performance with ablation experiments on the MovieLens datasetand Amazon dataset. As shown in Table 6, the parameter $N_{c}$ denotes the number of the cross-modal fusion layers stacked in the module, and 0 denotes that the cross-modal fusion module is not in use. We have the following observations from Table 6: first, with the addition of cross-modal fusion module, the recommendation performance improves at least 0.5% in terms of AUC and 0.48% in terms of GAUC on the MovieLens dataset (0.41% and 0.56% on the Amazon dataset). Second, when the $N_{c}$ is not exceeding 3, the recommendation performance improves with the increase in $N_{c}$. The highest increase reaches 0.86% in terms of AUC and 0.87% in terms of GAUC on the MovieLens dataset (1.19% and 1.36% on Amazon dataset). Third, when the $N_{c}$ is more than 3, the recommendation performance drops instead. The possible reason is that the dimension of the extracted features is too high to remain low-level features.

#### 4.4.2. Evaluation of Model Efficiency

**Figure 4:**
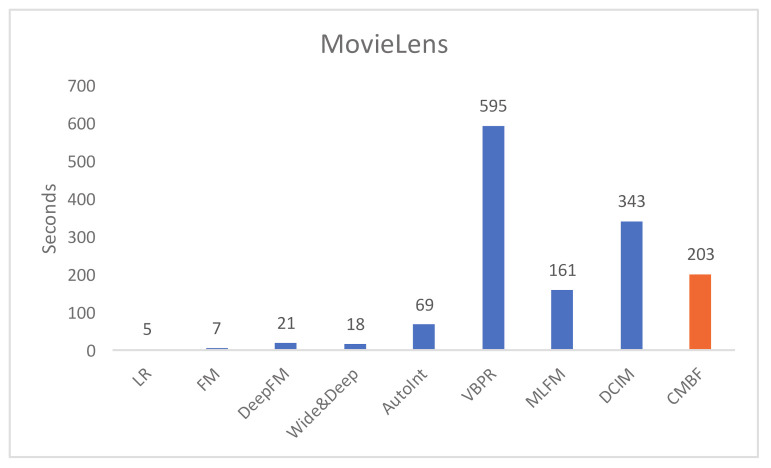
Run time per epoch of different algorithms on the MovieLens dataset.

**Figure 5:**
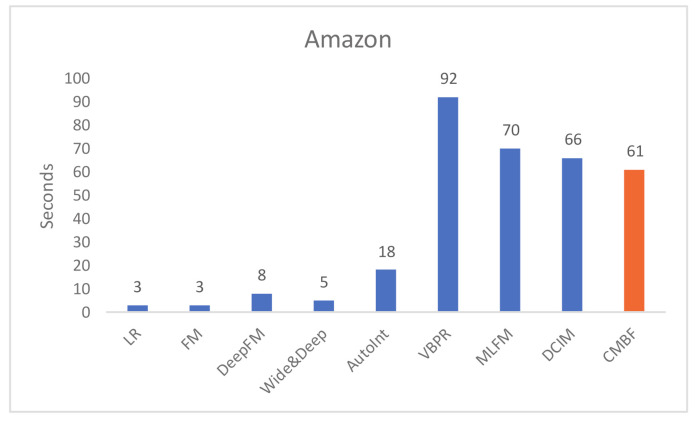
Run time per epoch of different algorithms on the Amazon dataset.

We compare the run time per epoch of CMBF and other algorithms, as shown in Figure 4 and Figure 5. Obviously, the run time of LR and FM is shortest because they do not use a deep network. The single-modal algorithms (i.e.; DeepFM, Wide&Deep, AutoInt) require less run time than multi-modal algorithms with worse performance of prediction results. Note that compared with other mult-modal algorithms (i.e.; VBPR, MLFM, DCIM), our CMBF does not require much run time, and its performance is improved, which proves the efficiency of CMBF.

## 5. Conclusions

In this paper, we proposed a Cross-Modal-Based Fusion Recommendation Algorithm (CMBF), which can alleviate the data sparsity problem in the recommendation system. The key to our algorithm is mining the relevance between two modalities and trying to obtain the high-level feature representation containing more information. Compared to existing multi-modal algorithms that use the simple fusion method, we propose the cross-modal fusion method to completely fuse the multi-modal features. We conduct experiments on two datasets and compared with other algorithms. The experimental results show that our proposed CMBF achieves the best recommendation performance. In addition, the ablation study proves that our cross-modal fusion method is an innovation in the multi-modal fusion field. However, the algorithm proposed in this paper is only suitable for the fusion of two modal features, and how to expand to three or more modal features requires further consideration.

## Figures and Tables

**Table 1:** Introduction of the MovieLens dataset and the Amazon dataset.

Dataset	User	Item	Interaction	Density
MovieLens	6040	3685	998,034	4.48%
Amazon	39,387	23,033	278,677	0.031%

**Table 2:** The processed attributes of the MovieLens dataset.

	Attributes	Dimension
User	Gender	2
	Occupation	21
	Age	61
	Zip Code	795
Movie	Type	19
	Year	37
	Image	2048
Others	Score Time	1

**Table 3:** The processed attributes of the Amazon dataset.

	Attributes	Dimension
User	ID	39,387
Product	Price	121
	TOP Sales	19
	Sales Rank	150
	Brand	157
	Type	1193
	Image	2048
Others	Score Time	1

**Table 4:** Comparison of different algorithms on the MovieLens dataset. The results in bold represent the best performances.

Algorithm	AUC	GAUC	Logloss
LR [25]	0.7775	0.5028	0.5441
FM [26]	0.7777	0.6004	0.5176
DeepFM [27]	0.7809	0.6837	0.4351
Wide&Deep [28]	0.7920	0.7239	0.4683
AutoInt [7]	0.8399	0.7712	0.3839
MiFiNN [29]	0.8772	-	0.3382
ADI [30]	0.8417	-	-
VBPR [12]	0.8419	0.7690	0.3700
MLFM [11]	0.8489	0.7789	0.3731
DCIM [10]	0.8655	0.7868	0.3665
CMBF	**0.8836**	**0.8363**	**0.3302**

**Table 5:** Comparison of different algorithms on the Amazon dataset. The results in bold represent the best performances.

Algorithm	AUC	GAUC	Logloss
LR [25]	0.4868	0.4793	0.3734
FM [26]	0.5137	0.4978	0.3524
DeepFM [27]	0.5625	0.5255	0.3426
Wide&Deep [28]	0.6264	0.5673	0.3390
AutoInt [7]	0.6415	0.5919	0.3296
VBPR [12]	0.6714	0.6053	0.3239
MLFM [11]	0.6862	0.6063	0.3179
DCIM [10]	0.7604	0.6092	0.3026
CMBF	**0.7880**	**0.6118**	**0.3001**

**Table 6:** Influence of parameter $N_{c}$ on the performance of CMBF. The results in bold represent the best performances.

	MovieLens			Amazon		
$N_{c}$	**AUC**	**GAUC**	**Logloss**	**AUC**	**GAUC**	**Logloss**
0	0.8750	0.8276	0.3417	0.7761	0.5997	0.3123
1	0.8800	0.8324	0.3352	0.7802	0.6053	0.3046
2	0.8831	0.8361	0.3312	0.7848	0.6087	**0.2998**
3	**0.8836**	**0.8363**	**0.3302**	**0.7880**	**0.6118**	0.3001
4	0.8824	0.8357	0.3321	0.7875	0.6072	0.3038
5	0.8813	0.8327	0.3333	0.7866	0.6028	0.3095

## Data Availability

The data presented in this study are available on request from the corresponding author.
